# Evaluation of Anti-Mutated Citrullinated Vimentin Antibodies, Anti-Cyclic Citrullinated Peptide Antibodies and Rheumatoid Factor in Omani Patients with Rheumatoid Arthritis

**DOI:** 10.1155/2012/285854

**Published:** 2012-08-15

**Authors:** Ahmed Al-Shukaili, Saif Al-Ghafri, Safia Al-Marhoobi, Juma Alkaabi

**Affiliations:** ^1^The Research Council of Oman, P.O. Box 1422, Muscat 130, Oman; ^2^Department of Medicine, College of Medicine and Health Sciences, Sultan Qaboos University (SQU), Muscat 130, Oman; ^3^Department of Studies & Planning, Statistics Section, The Research Council, Muscat 130, Oman

## Abstract

Rheumatoid factor (RF) is currently used in the diagnosis of rheumatoid arthritis (RA). The discovery of anticitrullinated protein autoantibodies has led to the development of various new tests, such as anti-cyclic citrullinated peptide (anti-CCP) antibodies, and anti-mutated citrullinated vimentin (anti-MCV) antibodies, to diagnose RA. The aims of this study were to determine the sensitivity and specificity of anti-MCV antibodies in comparison with anti-CCP antibodies and RF in Omani Arab patients with RA and compare our findings with published values from different ethnic groups. The sensitivity of anti-MCV antibodies was 72% with 87% specificity. For anti-CCP antibodies the sensitivity was 52% and the specificity was 97%. The sensitivity of RF was 57% with 94% specificity. Anti-CCP antibodies have higher diagnostic specificity and positive predictive value than RF and anti-MCV antibodies. Anti-MCV antibodies have the highest sensitivity when compared to anti-CCP antibodies and RF. Anti-MCV antibodies do not appear to be very useful in the diagnosis of RA. However, long-term study is required to find out whether anti-MCV antibodies can be used as predictive test for incidence of RA.

## 1. Introduction

Rheumatoid arthritis (RA) is a chronic systemic inflammatory autoimmune disease characterized by chronic joint inflammation that often leads to destruction of bone and cartilage, as well as the presence of autoantibodies including rheumatoid factor (RF) and highly RA-specific anti-cyclic citrullinated peptide (anti-CCP) antibodies [1].

RF and anti-CCP antibodies have been shown to be present prior to the appearance of clinical symptoms of arthritis suggesting that the initial immune dysregulation in RA occurs years before symptomatic disease [2]. Moreover, anti-CCP has been shown to be a specific prognostic marker for RA and predict the erosive or nonerosive progression of the disease. Thus, it is a useful tool for the optimal therapeutic management of RA patients [2–4]. 

Recently, anti-mutated citrullinated vimentin (anti-MCV) antibodies have been recommended to be better diagnostic marker for early arthritis [5]. Several studies demonstrated that anti-MCV antibodies have the same specificity as anti-CCP antibodies, but with better sensitivity [6–8]. Sghiri et al. (2008). showed that anti-MCV antibodies have a comparable sensitivity but lower specificity than anti-CCP antibodies, and concluded that anti-MCV antibodies do not appear to be very useful in the diagnosis of RA [7]. Moreover, a significant correlation has been established between anti-MCV antibody titers and both the severity of RA and the disease-activity score (DAS) [8]. Like anti-CCP antibodies, anti-MCV antibodies are also suitable for the early diagnosis of RA, with comparable sensitivity (55.3% versus 59.3%, resp.), specificity (92.1 versus 92.3%, resp.), and positive predictive value (95.8% versus 96.1%, resp.) [8]. 

Another study found that, in contrast to anti-CCP-positive patients, anti-MCV-positive patients exhibited significantly lower reduction in disease activity (DAS28) and a greater number of swollen joints [9, 10]. Thus, it appears that, anti-MCV antibodies may have the advantage of correlating better with disease activity and patient outcome than anti-CCP antibodies. 

The aims of this study were to determine the sensitivity and specificity of anti-MCV antibodies in comparison with anti-CCP antibodies and RF in Omani Arab patients with RA and compare our findings with published values from different ethnic groups.

## 2. Materials and Methods

### 2.1. Subjects

A total of 80 consecutive patients (71 female and 9 male, mean age 41.6 ± 14.5), attending outpatient clinic were randomly recruited in this study. All patients fulfilled the American College of Rheumatology (ACR) criteria for RA [11]. Patients with other rheumatic disease were excluded from this study.

 A total 133 healthy volunteers (70 female and 63 male, mean age 35 ± 7) were enrolled in this study. Those normal controls were obtained from Omani healthy workers at SQUH and College of Medicine, with no history of connective tissue disease, chronic infection/inflammation, cancer, or organ failure. Patients and control are sex (but not age) matched. A written informed consent was obtained from all participants. The study was approved by the local ethics committee.

Five milliliters of blood was drawn from the patients and the controls, into plain vacutainer tubes and sera was obtained by certification and stored at −20 until the time of the test.

Presence of RF was determined by the nephlometric method. ELISA techniques were used to detect anti-CCP antibodies (EUROIMMUNE, Medizinische Labordiagnostika, AG, Lubeck, Germany), and anti-MCV antibodies (ORGENTEC, Diagnostika GmbH, Mainz, Germany). The cut-off values of RF, anti-CCP antibodies, and anti-MCV antibodies were 30 U/mL, 5 RU/mL, and 20 U/mL, respectively. All those values were recommended by the manufacturers. 

### 2.2. Statistical Analysis

Data analysis was performed using SPSS version 20 software (SPSS Inc., Chicago, IL, US). The association between the categorical variables was tested using Chi-square test. Because the data was not normally distributed, a nonparametric test was used and the medians with interquartile range are presented. To test whether the medians of two unpaired sets of measurement are different from each other, we used the Mann-Whitney test. The level of significance at *P* < 0.05 was taken at 95% confidence interval (CI). 

## 3. Results

Table 1 shows the demographic information and some laboratory tests of RA patients and control groups. Of 80 patients with RA, 58 patients were positive for anti-MCV antibodies (72.5%), 49 patients were positive for anti-CCP antibodies (61%), and 47 patients were positive for RF (59%). By contrast, of 133 healthy controls, 16 persons were positive for anti-MCV antibodies (12%), 3 persons were positive for anti-CCP antibodies (2.3%), and 7 persons were weakly positive for RF (5%) (Table 1).

Anti-CCP antibodies showed the highest specificity (0.97) when compared to RF (0.94) and anti-MCV antibodies (0.87), with positive predictive value of 0.93 (Table 2). However, anti-MCV antibodies showed the highest sensitivity (0.72) when compared to anti-CCP antibodies (0.52) and RF (0.57). The sensitivity (but not specificity) of anti-MCV antibodies was significantly different (*P* = 0.023) when compared to the sensitivity of anti-CCP antibodies or RF.

As shown in Table 3, of 80 patients with RA, 49 patients were positive for anti-CCP antibodies, and 31 patients had negative anti-CCP antibodies. Of 49 RA patients with positive anti-CCP antibodies, 45 (92%) were anti-MCV antibodies positive, including only 6 patients (12%) with negative RF. Of 4 patients with positive anti-CCP antibodies and negative anti-MCV antibodies, 3 patients tested negative for RF. Of 31 RA patients with negative anti-CCP, 13 patients (42%) were positive for anti-MCV, including 7 patients (22.5) who tested negative for RF. Thus, anti-MCV was the only positive marker, for this group, indicating that anti-MCV assay may help to diagnose RA in patients with negative tests for anti-CCP and RF, Table 3. 

As shown in Table 4, we have correlated the levels of anti-MCV and anti-CCP antibodies with ESR and CRP; no significant correlations between these variables were observed, except between RF and ESR, where a significant correlation was obtained (*P* value = 0.011).

In Table 5, we have compared our findings of the sensitivity and specificity of anti-MCV, anti-CCP, and RF with other reported values from different countries. There was a significant difference in the sensitivity of the anti-MCV antibodies (*P* value = 0.04), among 5 studies included (7, 12–14); this difference can be attributed to the variation in the techniques used in those studies. However, in terms of specificity of anti-MCV, anti-CCP antibodies, and RF, no significant differences among those findings were observed. 

## 4. Discussion

The main aim of this study was to determine the sensitivity and specificity of anti-MCV antibodies, anti-CCP antibodies, and RF in Omani patients with RA. We found that anti-MCV antibodies have the highest sensitivity, and anti-CCP antibodies have the highest specificity. 

In recent years, many studies have evaluated the presence of anti-MCV, anti-CCP antibodies, and RF in RA patients. Marina et al. (2010), conducted a study with the Malaysian ethic group, which showed that the sensitivity of RF was higher than the sensitivity of anti-CCP or anti-MCV antibodies [12]. Our findings, however, showed that the sensitivity of anti-MCV antibodies was the highest. These differences can be attributed to two main reasons, one reason may be difference in the methods used in both studies; we have used a nephelometric method to measure RF, whereas Marina et al. used an ELISA technique. Other reasons may be due to genetic differences. However, both of these studies showed anti-CCP antibodies to have higher diagnostic specificity and positive predictive value than RF and anti-MCV antibodies [12]. Moreover, our finding were in agreement with several reports; Liu et al. 2009 showed that the sensitivities of anti-MCV antibodies, anti-CCP antibodies and RF were 78.2%, 61.8%, and 72.4%, respectively [13]. 

Our study also showed anti-CCP antibodies have a higher odd ratio for the prediction of developing RA, compared to anti-MCV antibodies or RF, indicating that anti-CCP antibodies may be a better prognostic indicator. However, this does not mean that anti-CCP can replace RF in diagnostic and prognostic testing for RA. Hence we found that, 22.5% of anti-CCP-negative patients were positive for RF, which is comparable with previous reports [14–18].

It has been documented that anti-MCV antibodies have a higher sensitivity and also a better prognostic marker for future radiographic changes than anti-CCP antibodies and RF [13, 14]. It has also been reported that anti-MCV antibodies were correlated with disease activity parameters such as DAS28, erythrocyte sedimentation rate (ESR), serum C-reactive protein (CRP) levels, serum RF levels, tender joint and swollen joint number [14–18]. In addition to their optimal specificity and ability to distinguish between erosive and nonerosive disease both anti-CCP and anti-MCV antibodies are present in very early stages of RA [14–19]. Unfortunately, the disease activity (DAS28) could not be obtained due to missing patient information such as number of swollen/tender joints. Another, limitation of this study was the random selection of our RA patient. We could not differentiate early arthritis from late arthritis, because new patients were referred from regional hospital (primary hospitals) to our clinic (tertiary hospital) after the onset of disease and this process may take up to one year. However, we have correlated anti-MCV, anti CCP, RF, ESR, and CRP levels and observed no correlation between these parameters except for a correlation between RF and ESR (Table 4).

In conclusion, our results for anti-MCV antibodies were comparable with other reported results with different ethnic groups. Generally, anti-MCV antibodies do not appear to be a very useful diagnostic test for RA, when compared to anti-CCP. Long term (5–7 years) monitoring of healthy controls with high titer of anti-MCV antibodies is recommended to assess the predictive value of anti-MCV antibodies.

## Figures and Tables

**Table 1:** Demographic information and laboratory investigations of Rheumatoid Arthritis (RA) patients and control groups.

Variables	RA patients	Healthy control	*P* value
	*N* = 80	*N* = 133	
Age (year, mean ± SD)	41.6 ± 14.5	35 ± 7	0.875
Sex (%)			
Female	71 (87.5)	70 (52.6)	0.983
Male	9 (12.5)	63 (47.4)	
Smoking status (%)			
Yes	1 (1.2)	2 (1.5)	0.28
No	79 (99)	131 (98.5)	
Disease duration (year, mean ± SD)	10.3 ± 18.7	NA	
ESR median (interquartile range)	23 (28)	NA	
CRP median (interquartile range)	8 (16)	NA	
Positive (%)			
Anti-MCV (>20 U/mL)	58 (72.5)	16 (12)	<0.0001
Anti-CCP (>5 RU/mL)	49 (61)	3 (2.3)	<0.0001
RF (>30 U/mL)	47 (59)	7 (5)	<0.0001

NA: not available, ESR: erythrocyte sedimentation rate, CRP: C-reactive protein, MCV: mutated citrullinated vimentin, CCP: cyclic citrullinated peptide, and RF: rheumatoid factor.

**Table 2:** Sensitivity and specificity of anti-MCV antibodies, anti-CCP antibodies, and RF in RA patients.

Test	Sensitivity (95% CI)	Specificity (95% CI)	Positive predictive value (95% CI)	Negative predictive value (95% CI)	Odd ratio
Anti-MCV	0.72 (65.3–78.6)	0.87 (82.8–90.2)	0.78 ( 71.8–80.4)	0.83 (79.9–86.5)	0.726
Anti-CCP	0.52 (49.5–63.2)	0.97 (91.7–96.8)	0.93 (78.7–90.9)	0.76 (71.9–79.7)	4.2
RF	0.57 (52.7–60.3)	0.94 (88.9–92.6)	0.87 (76.5–86.6)	0.78 (74.3–82.8)	1.9

Anti-MCV: mutated citrullinated vimentin, anti-CCP: anti-cyclic citrullinated peptide, RF: Rheumatoid factor, and RA: rheumatoid arthritis.

**Table 3:** Distribution of the rheumatoid arthritis patients according to the presence of anti-MCV, anti-CCP, and IgM-RF.

	Anti-CCP positive (>5 RU/mL)	Anti-CCP negative (≤5 RU/mL)	*P* value
	*n* = 49 (%)	*n* = 31 (%)	
Anti-MCV			
Positive (>20 U/mL)	45 (92)	13 (42)	<0.0001
Negative (≤20 U/mL)	4 (8)	18 (58)	
RF+ (>30 U/mL)			
Anti-MCV+	37 (75.5)	6 (19)	0.55
Anti-MCV−	3 (6)	1 (3)	
RF− (≤30 U/mL)			
Anti-MCV+	6 (12)	7 (22.5)	0.05
Anti-MCV−	3 (6)	17 (55)	

Anti-CCP: anti-cyclic citrullinated peptide, anti-MCV: anti-mutated vimentin citrullinated antibody, and RF: rheumatoid factor.

**Table 4:** Correlation analysis of anti-MCV antibodies, anti-CCP antibodies, RF, ESR, and CRP.

Test	ESR	CRP
Anti-MCV	0.358	0.472
Anti-CCP	0.452	0.575
RF	**0.011** ^ ∗^	0.833

ESR: erythrocyte sedimentation rate, CRP: C-reactive protein, MCV: mutated citrullinated vimentin, CCP: cyclic citrullinated peptide, RF: rheumatoid factor. ^∗^Significant *P* value.

**Table 5:** Comparison of sensitivity and specificity of anti-MCV, anti-CCP, and RF from different countries.

	Malaysia [12]	Netherlands [20]	Turkey [21]	Tunisia [7]	Oman	*P* value
Sensitivity (%)						
Anti-MCV	80	57	49.8	74.1	72	0.04
Anti-CCP	71	50	60.2	72.4	52	0.13
RF	80	47.7	67.8	65.9	57	0.055

Specificity (%)						
Anti-MCV	59.5	78.4	91.6	79	87	0.106
Anti-CCP	94.8	88.4	98.8	96.1	97	0.95
RF	74.5	86.1	91.6	74.4	94	0.39

Anti-MCV: mutated citrullinated vimentin, anti-CCP: anti-cyclic citrullinated peptide, RF: Rheumatoid Factor, RA: rheumatoid arthritis.
